# Medical statistics education in the big data era: focusing on critical thinking and ethical competence

**DOI:** 10.3389/fpubh.2026.1847906

**Published:** 2026-08-12

**Authors:** Yan Gao

**Affiliations:** Institute of Artificial Intelligence in Sports, Capital University of Physical Education and Sports, Beijing, China

**Keywords:** data ethics, data standardization, education framework, medical big data, medical statistics, normal distribution

## 1 Introduction

In the big data era, medical research is experiencing a fundamental change from small-sample validation to large-sample forecasting, requiring the combination of traditional statistical theories and cutting-edge data processing technologies. As a link connecting data with clinical application, medical statistics is vital for explaining disease pathogenesis, improving treatment plans, and establishing public health policies (1). Among traditional statistical models, the normal distribution, also known as Gaussian distribution, is broadly used in medical studies because it fits many physiological indexes such as blood pressure, blood glucose and body temperature (2). For example, the reference intervals of common clinical indexes, such as 3.9–6.1 mmol/L for adult fasting blood glucose, are mainly built on the rules of normal distribution (3). The widely recognized gold-standard practices are listed as follows: Shapiro–Wilk test for normality assessment of small samples, AD test and D'Agostino test for large samples, together with Q–Q plots for visual inspection. *Z*-transformation is the standard normalization approach, the 3σ criterion is applied to detect outliers, and normal-distribution-based methods are adopted to calculate medical reference ranges, laying the prerequisite foundation for parametric analyses including *t*-test and ANOVA (4, 5). Traditional analytical routines strictly rely on normality assumptions and face limitations when coping with heterogeneous high-dimensional medical big data. Differently, our work elaborates the complementary relationship between normal distribution and machine learning as well as nonparametric models, and further constructs an ethics-embedded teaching framework integrating statistical theories, big data applications and data ethics education, aiming to advance medical statistics teaching and translational clinical data analysis. Nevertheless, conventional analysis based on normal distribution has natural defects in the big data background: small samples will result in untrustworthy deduction of population features (6). On the other hand, medical big data, featured by large scale, diverse sources and high dimensions, often encounters the problem of “plenty of data but lack of information” without standardized statistical guidance (7). This shows that it is urgent to connect normal distribution with big data. The combination of the two is reasonable because they complement each other: first, the large sample of medical big data supports more precise fitting of the theoretical hypothesis of normal distribution, improving the dependability of statistical deduction (8); second, normal distribution offers a strict and standardized system for processing multi-type medical data such as electronic health records and gene data, which prepares for further big data exploration (9). There is no doubt that non-normal methods including machine learning algorithms and generalized linear models are highly applicable in medical big data analysis (10). Random forest and support vector machine perform well in dealing with high-dimensional and non-linear data such as gene sequencing data, while logistic regression and Poisson regression are often used for discrete result variables like disease onset by giving up the requirement of normality (11). But these approaches cannot take the place of normal distribution; instead, they build a supplementary analysis system together. For instance, in the prediction of type 2 diabetes, normal distribution is first used to unify the scales of blood glucose and blood lipid, removing unit differences, and then machine learning models are used to raise prediction precision (12). This complementary relation further stresses the significance of combining normal distribution with big data technologies. The integration of normal distribution and big data also puts forward new requirements for medical statistics education: learners should master not only the basic theory of normal distribution and big data tools but also the ethical influence of data use such as privacy protection and health fairness (13). Existing models include lecture-based, problem-based learning (PBL), case-based learning (CBL) and blended learning, mostly focusing on statistical computation (14). Few systematically incorporate data ethics. Our framework integrates normal distribution theory, big data practice and ethical education, forming a more comprehensive training system. This framework was developed on the basis of widely recognized pedagogical models including PBL, CBL and blended learning reported in medical statistics education literature, alongside long-term accumulated practical experience from our classroom teaching practice (15). Consistent with prior reforms, it adopts student-centered modes to improve statistical practical ability. Differently, existing studies seldom systematically integrate normal distribution theory, medical big data analysis and data social responsibility ethics, while our design fills this targeted gap (14).

Traditional medical statistics education pays more attention to teaching theoretical knowledge, without systematic training on data integration and social responsibility (16). To solve this shortage, this study constructs an ethics-included teaching model that combines normal distribution, big data application and social responsibility training. The main goals are: (1) to help learners grasp the methods of combining normal distribution and big data; (2) to develop critical thinking on data ethics; (3) to strengthen social responsibility in using medical data. This research aims to offer new thoughts for promoting medical statistics education and boosting the transformation of medical big data into clinical evidence.

## 2 Normal distribution in medical statistics: a foundational framework for big data integration

Normal distribution, distinguished by its symmetric profile, central mean, and standardized deviation, represents a cornerstone statistical model across medical research. A multitude of physiological phenotypes—including height and body weight—and critical disease risk factors, such as circulating cholesterol levels, exhibit normatively distributed patterns. This inherent conformity empowers investigators to define clinically valid reference intervals, execute robust statistical inference, and rigorously evaluate the therapeutic efficacy of novel pharmaceutical agents (17).

### 2.1 Application of normal distribution in standardizing human health data

Continuous quantitative indicators constitute the core data basis of population health analysis, and standardized data processing is essential to guarantee consistent statistical inference and clinical applicability. Normal distribution serves as a fundamental statistical tool for standardizing such continuous health metrics in medical big data research. Taking hypertension population analysis as a typical scenario, standardized Gaussian-based analytical procedures can systematically process large-sample blood pressure data, effectively differentiating normal population baseline ranges from clinically meaningful abnormal thresholds and supporting objective disease screening and population health evaluation (18). Beyond single clinical indicators, modern medical research integrates multi-source heterogeneous data including electronic health records, wearable monitoring information and geospatial environmental variables. Normal distribution modeling enables unified normalization of core physical and clinical indicators such as BMI, constructing a standardized analytical framework for exploring the genetic and environmental influencing factors of chronic diseases (19). Notably, medical datasets commonly present inherent skewness and non-Gaussian characteristics, exemplified by the uneven distribution of hypertension onset age. For such non-normal variables, targeted logarithmic transformation and log-normal distribution fitting can optimize data distribution characteristics, improving the robustness, accuracy and interpretability of subsequent statistical analysis (20).

### 2.2 Normal distribution as a pillar of medical big data preprocessing

Medical big data aggregated from multi-level medical institutions typically features heterogeneous formats, inconsistent calibration standards and sporadic abnormal noise, making standardized preprocessing a prerequisite for credible statistical analysis. Normal distribution theory serves as a core foundational paradigm for addressing such data heterogeneity and quality fluctuation in medical datasets. Through Gaussian distribution-based screening, anomalous outlier records in clinical indicators such as blood glucose can be effectively identified and corrected, which minimizes systematic errors caused by inconsistent data scales and unit differences across different medical platforms. Combined with modern big data analytical techniques, normality-based standardized modeling supports continuous, dynamic monitoring of individual health trajectories in intelligent medical systems, significantly improving the sensitivity for early identification of subtle pathological changes and disease progression risks (21). In chronic disease risk assessment, standardized normalization of longitudinal blood glucose indicators enables accurate trend quantification and stable long-term risk prediction for diabetes, realizing robust data-driven medical early warning (22).

The generalized applicability of normality-oriented standardized analysis in biomedical research is further validated in multi-dimensional medical imaging data processing. Taking brain magnetic resonance imaging (MRI) quantitative analysis as a typical application scenario, normal distribution modeling can establish unified normative reference intervals for macroscopic and microscopic anatomical characteristics of brain tissues, enabling precise quantitative evaluation of individual structural deviations (23). This standardized modeling strategy effectively compensates for the poor replicability and low stability inherent in traditional small-sample biomedical models, providing reliable translational analytical support for neurological disease detection and comparative medical research.

Importantly, competent medical data analysis requires not only proficient application of normality-based standardized workflows, but also critical awareness of the applicable boundaries of Gaussian assumptions, which constitutes a key component of the statistical literacy cultivated in this study's teaching system. Real-world biomedical data, including sparse omics sequencing results, clinical counting indicators and rare disease statistical records, frequently presents zero-inflated characteristics and long-tailed skewed distribution, which fundamentally deviates from standard Gaussian distribution (24). Blind application of conventional parametric statistical methods including *t*-tests, ANOVA and linear regression under non-normal conditions will substantially distort analytical results and lead to biased clinical interpretation (25). Therefore, this teaching framework emphasizes mandatory normality verification in preliminary data analysis, and guides students to flexibly adopt targeted correction strategies including data transformation, zero-inflated statistical models and non-parametric testing according to actual data distribution characteristics (26). This training mode avoids mechanical, formulaic statistical application, and effectively cultivates students' critical thinking and rigorous analytical competency for complex and heterogeneous medical big data.

## 3 An ethics-embedded pedagogical framework: integrating normal distribution, big data literacy, and social responsibility

Social responsibility is defined as ethical awareness throughout the full lifecycle of medical data use, encompassing patient privacy preservation, prevention of analytical bias, and responsible translation of statistical findings into credible clinical evidence. This section proposes a systematic pedagogical framework that organically unifies normal distribution statistical methodology, professional big data literacy, and sustainable social responsibility cultivation, aiming to address the disconnection between technical statistical training and ethical governance in conventional medical statistics education.

### 3.1 Core educational objectives

The ethics-infused pedagogical structure is engineered to accomplish three interrelated core objectives covering statistical technique, big data literacy, and social responsibility: (1) professional competency: equip learners with in-depth mastery of the theoretical underpinnings of the normal distribution and its translational integration with big data analytics in clinical and public health contexts, so as to solidify fundamental big data literacy for standardized medical data analysis. (2) Ethical accountability: enhance critical awareness of normative and ethical challenges in medical big data deployment, including data privacy safeguarding and health equity promotion. (3) Critical thinking: cultivate balanced, multi-perspective reasoning regarding the dynamic tension between technological innovation and ethical governance in data-driven medicine (27).

### 3.2 Curriculum modules

**Figure 1:**
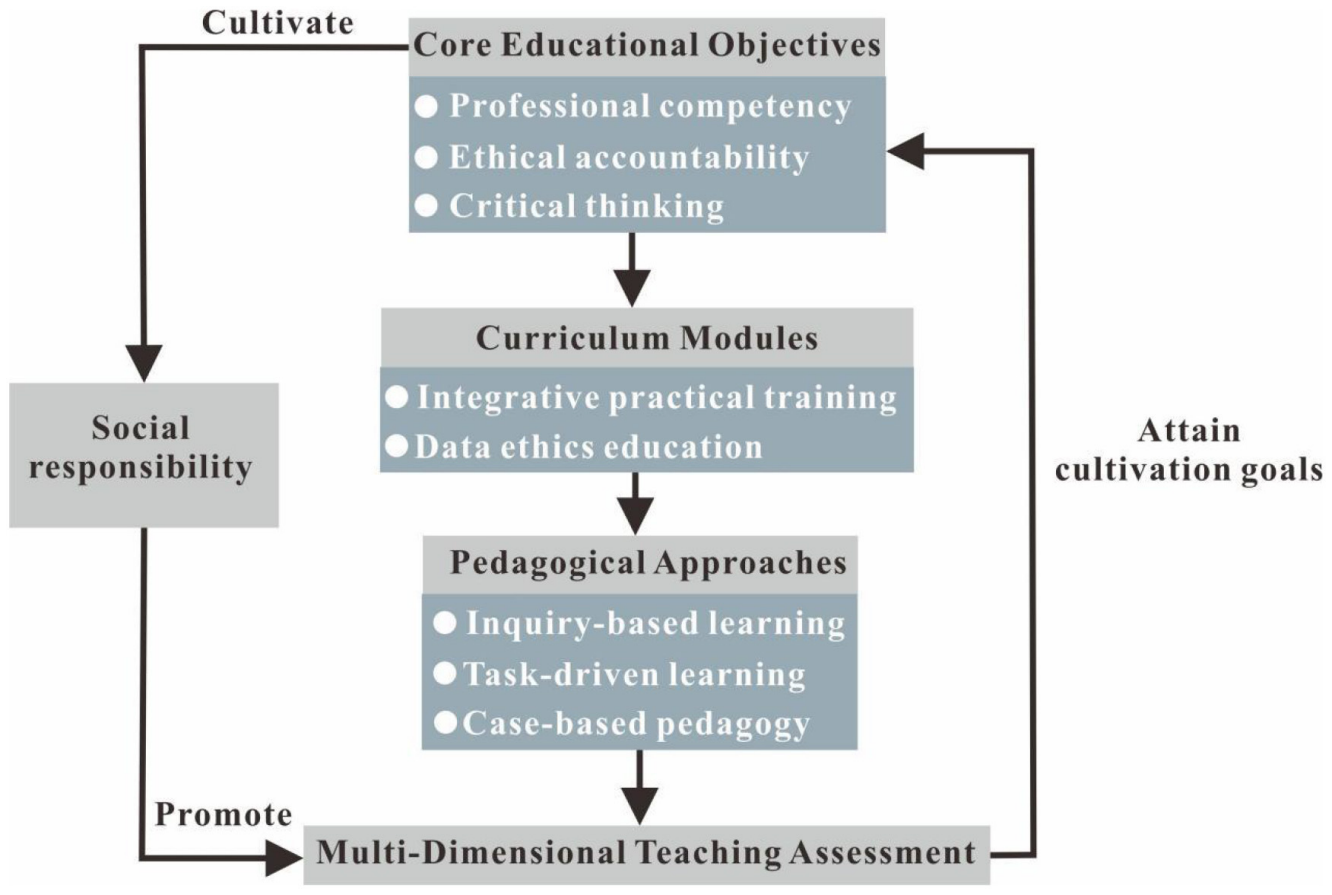
Schematic diagram of the education model.

The curriculum is structured around two interconnected core modules (Figure 1):

Integrative practical training: normal distribution-guided big data preprocessing pipelines—including data standardization, outlier filtering, and dimensional homogenization—supplemented with translational case studies such as hypertension risk stratification via joint normal distribution and big data modeling (28). (2) Data ethics education: regulatory frameworks governing health data privacy, ethical dilemmas in big data-enabled population health analysis, and real-world cases of inappropriate data utilization and its societal ramifications (29). This module complements technical training by shaping students' social responsibility to ensure compliant, equitable, and credible medical data interpretation and application.

### 3.3 Pedagogical approaches

Three interactive, learner-centered instructional modalities are implemented:

(1) Inquiry-based learning: facilitate structured deliberation on pivotal normative questions, such as balancing scalable data utilization and individual privacy protection in normal distribution-based big data analytics, to deepen ethical reasoning (30). Students conduct structured group debates on the ethical trade-off between large-scale normality-test-driven medical big-data mining and individual patient privacy protection, practicing systematic ethical reasoning throughout discussion (31).
(2) Task-driven learning: in hands-on projects analyzing weight and blood glucose datasets for personalized health interventions, students must complete standardized ethical checklists to mitigate selection bias, avoid unauthorized data disclosure, and guarantee compliant application of normal distribution analytical methods (32). (3) Case-based pedagogy: analyze high-impact real-world incidents involving unauthorized health data access or misuse to illustrate tangible societal harms arising from ethically unsound data practices (33). We adopt real public incidents of unauthorized health-data access and statistical misuse, guiding students to analyze concrete ethical violations, quantify resultant social harms, and summarize standardized data governance protocols (34).

### 3.4 Multi-dimensional teaching assessment

This study constructs a holistic multi-dimensional evaluation system for medical statistics teaching, which comprehensively assesses students' comprehensive literacy of statistical application, practical operation and data ethics from three core dimensions (35). The theoretical evaluation focuses on students' systematic mastery of normal distribution core theories and their comprehensive understanding of the integration logic between classical statistical principles and medical big data analytical frameworks, adopting a combination of quantitative scoring and qualitative capability judgment. The practical evaluation centers on real medical data analysis scenarios, aiming to examine students' practical proficiency in core operational skills including normality-based data standardization, big data preprocessing and model applicability verification. Complementing theoretical and practical assessments, the ethical evaluation targets students' comprehensive ability to identify, analyze and properly address potential ethical risks in medical data research through case analysis and scenario simulation training. To fully verify the overall teaching effectiveness and ensure the achievement of preset training objectives, diversified summative assessment methods including classroom performance observation, standardized practical task reports and teaching reflection essays are integrated into the whole teaching evaluation process.

## 4 Conclusion

This study explores the sustainable application of normal distribution theory in medical big data analysis and develops a multi-dimensional pedagogical framework integrating statistical practice, critical thinking, and data ethics. Different from the prevalent view that classical statistics is outdated in big data contexts, normal distribution remains irreplaceable for standardized medical data calibration, heterogeneous data unification, and interpretable clinical inference, and it can be well combined with machine learning and non-parametric models to optimize analytical reliability. Critically, Gaussian assumptions are not universally applicable, as zero-inflated and long-tailed biomedical data may cause analytical bias through rigid usage. This framework avoids mechanical statistical application and adapts to intelligent medical analysis demands. Limited by the research scope, this study lacks long-term quantitative teaching effect verification. Future work will optimize targeted modules for non-normal data and improve longitudinal evaluation systems, providing a feasible reference for medical statistics teaching reform.
